# Divergence in Life History Traits between Two Populations of a Seed-Dimorphic Halophyte in Response to Soil Salinity

**DOI:** 10.3389/fpls.2017.01028

**Published:** 2017-06-16

**Authors:** Fan Yang, Jerry M. Baskin, Carol C. Baskin, Xuejun Yang, Dechang Cao, Zhenying Huang

**Affiliations:** ^1^State Key Laboratory of Vegetation and Environmental Change, Institute of Botany, Chinese Academy of SciencesBeijing, China; ^2^Institute of Sericulture, Chengde Medical UniversityChengde, China; ^3^Department of Biology, University of Kentucky, LexingtonKY, United States; ^4^Department of Plant and Soil Sciences, University of Kentucky, LexingtonKY, United States

**Keywords:** halophyte, plant life history traits, population divergence, reproductive allocation, seed germination, seed heteromorphism, seed morph ratio

## Abstract

Production of heteromorphic seeds is common in halophytes growing in arid environments with strong spatial and temporal heterogeneity. However, evidence for geographic variation (reflecting local adaptation) is almost nonexistent. Our primary aims were to compare the life history traits of two desert populations of this halophytic summer annual *Suaeda corniculata* subsp. *mongolica* and to investigate the phenotypic response of its plant and heteromorphic seeds to different levels of salt stress. Dimorphic seeds (F_1_) of the halophyte *S. corniculata* collected from two distant populations (F_0_) that differ in soil salinity were grown in a common environment under different levels of salinity to minimize the carryover effects from the field environment and tested for variation in plant (F_1_) and seed (F_2_) traits. Compared to F_1_ plants grown in low soil salinity, those grown in high salinity (>0.2 mol⋅L^-1^) were smaller and produced fewer seeds but had a higher reproductive allocation and a higher non-dormant brown seed: dormant black seed ratio. High salinity during plant growth decreased germination percentage of F_2_ black seeds but had no effect on F_2_ brown seeds. Between population differences in life history traits in the common environment corresponded with those in the natural populations. Phenotypic differences between the two populations were retained in F_1_ plants and in F_2_ seeds in the common environment, which suggests that the traits are genetically based. Our results indicate that soil salinity plays an ecologically important role in population regeneration of *S. corniculata* by influencing heteromorphic seed production in the natural habitat.

## Introduction

In arid and semi-arid regions of the world, soil salinity is an environmental stress factor to plants, partly because soil conditions are strongly heterogeneous in space and time (Bernstein, 1975). Much work on halophytic species has focused on their morphology, photosynthesis, physiology and molecular biology in an attempt to understand their distinctive adaptations to saline environments (Bernstein, 1975; Zhu, 2001; Redondo-Gómez et al., 2006; Pandolfi et al., 2012). Fluctuations in soil salinity potentially can result in high mortality in the germinating seed cohorts, and therefore high salt tolerance of seeds of halophytes during the germination stage has been studied (Ungar, 1978; Khan and Gul, 2006; Ameixa et al., 2016). As a link between two generations, seeds traits have a direct impact on population regeneration and dynamics (Harper, 1977; Donohue, 2009). Seed dormancy and germination responses enable seedling populations to emerge at the right time under relatively favorable environmental conditions that ensure establishment and completion of the life cycle (Hoyle et al., 2015; Huang et al., 2016). Therefore, seed traits and subsequent growth strategy are important for seedling survival and reproductive success in arid and saline habitats (Ungar, 1987; Gutterman, 2002; Yang et al., 2012).

Seed heteromorphism, i.e., the production by a single plant of two or more seed morphs that differ in mass, morphology, and ecology, is prevalent in arid habitats, and it is particularly common in halophytes (Khan and Ungar, 2001; Imbert, 2002). Harper (1977) hypothesized that seed heteromorphism confers a selective advantage to plants that grow in extreme, fluctuating environments, for example because different seed morphs may germinate at different times due to differences in dormancy and seed coat properties (Mohamed-Yasseen et al., 1994; Baskin and Baskin, 2014). A prolonged overall germination period enabled by the combination of different seed morphs may provide more opportunities for establishment of seedling cohorts than monomorphic morphs by spreading the risks of survival in variable environments (Venable, 1985a; Venable et al., 1987). Heteromorphic seeds not only have distinct dormancy/germination behaviors (Mandák and Pyšek, 1999; Wang et al., 2008; Yao et al., 2010; Cao et al., 2012; Baskin and Baskin, 2014), but plants growing from them can differ in various life history traits such as survival and biomass allocation (Sorensen, 1978; Mandák and Pyšek, 2005; Ruiz de Clavijo, 2005; Wang et al., 2012; Baskin and Baskin, 2014; Yang et al., 2015a). Thus, the relative production of different seed morphs can be used to predict germination behavior and adaptive strategy of a heteromorphic population (Venable et al., 1995).

Variation in seed (and seedling) traits of seed-heteromorphic halophytes occurs among populations (Ladiges et al., 1981; Song et al., 2008). However, evidence for geographic variation (reflecting local adaptation) is almost nonexistent probably because the carryover effects of field environments have not been ruled out in most studies. By investigating natural populations, any phenotypic differences between them can be distinguished. Further, by rearing plants from different habitats in a common environment under different levels of salinity, we can remove the effects of the natural (maternal) environment and test the phenotypic plasticity response of heteromorphic seeds to soil salinity. We previously reported that maternal soil salinity can affect heteromorphic seed germination and seedling growth (Yang et al., 2015b), but our knowledge of seed morph production is much lower than that of other life history traits influenced by soil salinity. Here, we hypothesized that (1) phenotypic differences occur between populations in the field; (2) these differences in plants and seed traits between populations are genetically based; and (3) seed morph production and germination behavior of heteromorphic seeds are influenced by the magnitude of soil salinity. We tested our hypothesis using two distant populations (about 1700 km away from each other in northern China) of the halophyte *Suaeda corniculata* (C. A. Meyer) Bunge subsp. *mongolica* Lomon. & Freitag (Amaranthaceae) (hereafter *Suaeda corniculata*), which differ strongly in habitat soil salinity regime.

*Suaeda corniculata* is a succulent summer annual herb that grows in saline-alkaline soils (Zhu et al., 2003) and is widely distributed in steppes and semiarid and arid zones of northern China, Mongolia and south-central Siberia (Lomonosova et al., 2008). It produces two diaspore morphs: brown and black seeds. The brown seeds are non-dormant, whereas the black seeds are dormant and can exhibit an annual dormancy/non-dormancy cycling in the soil seed bank (Cao et al., 2012). Dimorphic seeds differ in germination timing (seedlings derived from brown seeds emerged in spring and those from black seeds in summer), but seedling recruitment and survival percentages were almost the same (Cao et al., 2012; Yang et al., 2015a).

## Materials and Methods

### Field Site Description

**FIGURE 1:**
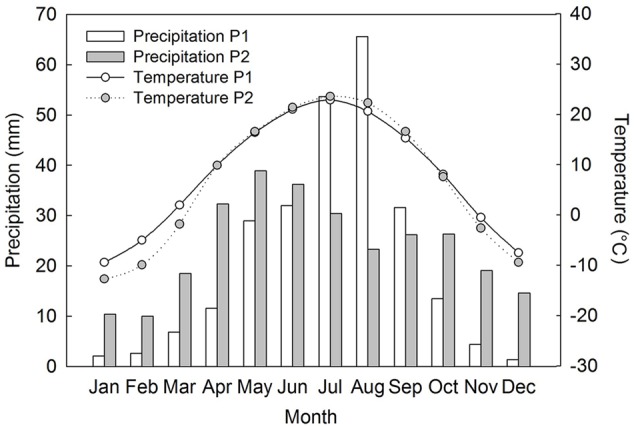
Recent 30 years of precipitation and mean air temperature data at the two field study sites of *Suaeda corniculata* (CMDSSS, 2015). P1, population 1; P2, population 2.

Both populations of *Suaeda corniculata* occur around shores of saline lakes. Population 1 (P1) is distributed in a saline steppe on the Ordos Plateau of Inner Mongolia, northcentral China (38° 14′ N, 107° 29′ E, 1311 m a.s.l.), where mean annual precipitation is 249.8 mm and mean annual temperature 8.2°C. Population 2 (P2) occurs in a cold desert of the Junggar Basin of Xinjiang, northwest China (43° 43′ N, 87° 37′ E, 429 m a.s.l). This area also has a semi-arid climate with a mean annual precipitation of 286.2 mm and a mean annual temperature of 6.9°C (CMDSSS, 2015). P1 has a distinct autumn/winter/early spring dry season with most of the precipitation occurring in late-spring/summer and peaking in July–August (CMDSSS, 2015). P2 has a similar precipitation pattern but with relatively more precipitation in autumn/winter/early spring and with rainfall peaking earlier in the summer than in P1 (**Figure 1**).

### *In Situ* Differences between the Two Study Populations

**FIGURE 2:**
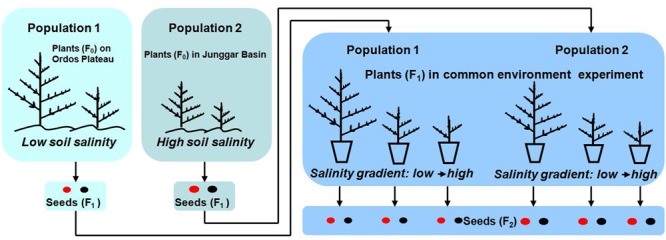
Experimental design of study.

This part of the study was done to verify (or not) differences in the two study populations in situ in order to better compare their responses to salt stress in a pot experiment (for experimental design of the study see **Figure 2**). Four soil cores (each 5 cm length × 5 cm width × 20 cm depth) were collected from P1 or P2 in mid-October (2010 and 2011 for P1; 2010 and 2012 for P2), and the soil cores were sub-divided in to four layers: 0–5 cm, 5–10 cm, 10–15 cm, 15–20 cm. Mean total soil salinity in the 0–20 cm soil layer were determined by soil salinities of the 16 soil samples (4 cores × 4 layers) by using the residue drying quality method (Bao, 2000). Twenty individual plants in each natural population were haphazardly selected to determined life history traits, including life span, plant height, reproductive allocation, seed morph ratio, seed size, and germination percentage.

### Common Environment Experiment

Mature seeds used in the experiment were collected from several hundred plants on 20 October 2012 from P1 and on 25 September 2012 from P2. Seeds were dried for 10 days at room conditions (10–18°C, 17–32% RH) and sorted into brown and black morphs. Then, the seeds were put into cloth bags and buried in soil at a depth of 2 cm near the experimental site until used in a common environment experiment, which was begun in spring 2013. Thus, the seeds were cold stratified during winter 2012/2013 before the experiments were begun in spring 2013.

The common environment experiment was conducted in two well-ventilated screen houses (one for P1 and the other for P2) at Ordos Sandland Ecological Research Station of the Chinese Academy of Sciences (39° 29′ N, 110° 11′ E, 1300 m a.s.l.). The houses were covered with sheets of plastic only during rains. Seeds of the two morphs from each population were sown into 12-cm-diameter × 20-cm-tall plastic pots on 20 May 2013. Pots were filled with vermiculite and sand (2 L, 1:1, v/v) to which was added 6 g of sustained release fertilizer (Osmocote 5#, Scotts, Marysville, OH, United States, with a 14 N: 13 P: 13 K elemental ratio, duration 5–6 months). Plants from P1 and P2 were kept in separate houses (about 32 m away from each other) to prevent cross pollination between them. Plants of each population were arranged randomly, and they were assumed to be pollinated by the same pollen pool of their population (Latzel et al., 2014), and the position differences of plants in house could be excluded.

Addition of salt was begun about three weeks after the sowing date, when all seedlings had established. Only NaCl was used to regulate soil salinity, because it is the major component of soil salinity in the study areas of the two populations (content of main ions in soil was determined by an inductive coupled plasma emission spectrometer, ICAP6300 (Dionex, Sunnyvale, CA, United States) and an ion chromatograph, ICS-1500 (Thermo Scientific, Waltham, MA, United States) in 2010). Pots received 500 mL of (1) tap water (0 mol⋅L^-1^ NaCl; control), (2) 0.1 mol⋅L^-1^ NaCl solution, (3) 0.2 mol⋅L^-1^ NaCl solution, (4) 0.4 mol⋅L^-1^, (5) 0.6 mol⋅L^-1^, or (6) 1.2 mol⋅L^-1^ once per week. To avoid the risk of drought stress, 200 mL of tap water were applied to each pot per week during spring and autumn and 300 mL per week during summer. Salt addition lasted for eight weeks, during which time soil salinity in the pots reached 0 mol⋅L^-1^, 0.2 mol⋅L^-1^, 0.4 mol⋅L^-1^, 0.8 mol⋅L^-1^, or 1.2 mol⋅L^-1^ in salt additions 1, 2, 3, 4, and 5, respectively. However, plants did not survive under a salinity of 1.2 mol⋅L^-1^, and thus we could not collect any data for this treatment. Therefore, by the end of 2013 we had produced 16 categories and 160 individuals of maternal plants: 2 populations × 2 seed morphs (from which maternal plants were produced) × 4 soil salinities × 10 replicates. Each plant was harvested when it turned yellow, and the seeds were beginning to disperse.

### Key Life History Stages of Plants Grown in Common Environment Experiment

Seedling stage, vegetative period, reproductive period and life span were recorded by monitoring the following phenological events for F_1_ seeds and F_1_ plants (see **Figure 2**): (1) germination; (2) emergence of first branch from main stem, which indicates seedlings have established; (3) opening of first flower, which indicates the shift from vegetative growth to reproductive growth; and (4) dispersal of seeds, which indicates seeds have matured. Thus, the seedling stage was from (1) to (2), the vegetative period (1) to (3), the reproductive period (3) to (4) and plant life span (1) to (4).

### Vegetative and Reproductive Traits of Plants Grown in Common Environment Experiment

Vegetative mass and reproductive mass (total seed mass per plant) were measured after harvest of F_1_ plants (see **Figure 2**). Each plant was separated into vegetative (shoots) and reproductive (seeds) parts. After oven-drying at 75°C for 48 h, vegetative parts were weighed using a Sartorius BP 221 electronic balance (Sartorius AG, Gottingen, Germany) (0.0001 g). Reproductive mass was determined after drying for 10 days in room conditions.

### Traits of Seeds Produced in Common Environment Experiment

One-thousand seeds (F_2_, see **Figure 2**) were randomly selected from each F_1_ plant to determine the proportion of brown seeds (number of brown seeds/total number of seeds; proportion of black seeds = 1 – proportion of brown seeds). If the number of seeds per plant was less than 1000, we counted all of them. We measured seed diameter and 1000-seed mass of each seed morph using a Nikon 80i dissecting microscope equipped with a micrometer (Nikon Corporation, Tokyo, Japan) and the Sartorius electronic analytical balance, respectively. The number of seeds was calculated as reproductive mass per plant divided by 1000-seed mass multiplied by 1000.

Germination percentage of the F_2_ seeds produced by these F_1_ experimental plants was assessed. There were 32 categories of seeds (2 seed morphs of offspring seeds × 16 categories of maternal plants, see above), 128 Petri dishes (32 categories of seeds × 4 replicates) and 3200 seeds (25 seeds per Petri dish). Fresh brown and black offspring seeds were incubated on Whatman No.1 filter paper in 5-cm-diameter Petri dishes moistened with distilled water at 10/25°C (12 h darkness/12 h fluorescent light). The germination test lasted 3 weeks, by which time germination percentages had leveled off. The criterion for germination was emergence of the radicle. Germination was monitored every 24 h, and germinated seeds were removed at each count. After the germination test, non-germinated seeds were tested for viability with tetrazolium (Baskin and Baskin, 2014), and the germination percentage was calculated as the number of germinated seeds divided by (25 – dead seeds) multiplied by 100%.

### Statistical Analyses

The effects of population (P), salinity (S) and seed morph (SM) on phenology, plant height, biomass and seed traits were analyzed using three-way ANOVA, and the effects of P and SM on the length of seedling stage were analyzed using two-way ANOVA. These statistical analyses were performed with SPSS Version 17.0 (SPSS Inc., Chicago, IL, United States), and data were square-root or arcsine transformed when necessary to meet assumptions of ANOVA. Tukey’s HSD test was used to determine differences (*P* < 0.05) between individual treatments. Independent-samples *t*-test was used to compare significance (*P* < 0.05) in field data between the two populations.

## Results

### *In Situ* Differences between the Two Study Populations

P1 soil was less saline than that of P2 (**Table 1**). Plants (F_0_) growing in their natural habitat (P1 vs. P2) differed in life history traits (life span, plant height and reproductive allocation) and in seed (F_0_) traits (brown seed proportion, seed diameter and germination percentage). Plants at P2 (more-saline site) were smaller and had higher reproductive allocation, higher brown seed proportion, larger seeds and lower germination percentage of black seeds than those at P1 (less-saline site) (**Table 1**).

**Table 1:** Variation in field between two natural populations of *Suaeda corniculata*.

	Population 1 (Ordos Plateau)	Population 2 (Junggar Basin)	
**Mean total soil salinity (%)**			
Year 1	2.5 ± 0.1	4.0 ± 0.3	
	(407 ± 33 mmol⋅L^-1^ NaCl)	(691 ± 55 mmol⋅L^-1^ NaCl)	
Year 2	2.4 ± 0.2	3.6 ± 0.5	
	(419 ± 9 mmol⋅L^-1^ NaCl)	(624 ± 55 mmol⋅L^-1^ NaCl)	
Life span (d)	149.7 ± 1.1	137.8 ± 0.8	^∗^
Plant height (mm)	561.8 ± 14.3	442.4 ± 11.7	^∗^
Reproductive allocation (%)	21.8 ± 0.8	24.8 ± 0.8	^∗^
Brown seed proportion (%)	20.4 ± 4.0	67.2 ± 2.3	^∗^
Seed diameter (mm)			
Brown	1.1 ± 0.0	1.4 ± 0.0	^∗^
Black	1.0 ± 0.0	1.2 ± 0.0	^∗^
Germination of fresh seeds (%)			
Brown	91.8 ± 2.7	97.0 ± 1.9	
Black	80.0 ± 5.2	35.0 ± 5.3	^∗^

*^∗^Significant difference (*P* < 0.05) between populations.*

### Key Life History States of Plants (F_1_) Grown in Common Environment Experiment

**FIGURE 3:**
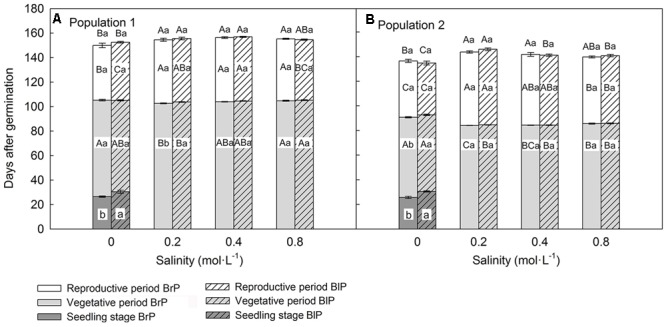
Effects of soil salinity and seed morph on length of key life history stages (mean ± 1 SE) of F_1_ (filial generation 1, see **Figure 2**) plants of the two populations of *Suaeda corniculata*. Different letters above bars indicate significant differences (*P* < 0.05) in length of life span and different letters within bars significant differences (*P* < 0.05) in length of seedling stage, vegetative period, and reproductive period. Uppercase letters indicate differences across all salinity levels in the same seed morph and lowercase letters differences between seed morphs for the same salinity level. BrP, plants from brown seeds; BlP, plants from black seeds. Addition of salt was begun after the seedlings had established, and data for seedling stage were collected under 0 mol⋅L^-1^ NaCl.

Population, salinity and their interactions all had a significant effect on the length of three key life history stages (vegetative period, reproductive period and life span), but seed morph had an effect only on length of the vegetative period (**Table 2**). The life span of P1 was longer than that of P2, and this was mostly due to P1 having a longer vegetative period than that of P2 (**Figures 3A,B**). However, the length of the reproductive period of P1 was shorter than that of P2 (**Figures 3A,B**). The length of vegetative period, reproductive period and life span was almost the same among different salinities (from 0.2 to 0.8 mol⋅L^-1^), except for 0 mol⋅L^-1^ (control), where plants had the longest vegetative stage but the shortest reproductive period and life span (**Figures 3A,B**). The seedling stage of plants from brown seeds was shorter than that for black seeds. This indicates that seedlings developed from brown seeds established more rapidly than those from black seeds (**Figures 3A,B**).

**Table 2:** Results of a two/three-way ANOVA on effects of population (P), salinity (S) and seed morph (SM) on life history traits of *Suaeda corniculata.*

		Seedling stage			Vegetative period			Reproductive period			Life span			Vegetative mass			Reproductive mass	
Source	df	*F*-value	*P*-value	df	*F*-value	*P*-value	df	*F*-value	*P*-value	df	*F*-value	*P*-value	B	*F*-value	*P*-value	df	*F*-value	*P*-value
Population (P)	1	0.15	0.697	1	7849.73	**<0.001**	1	45.13	**<0.001**	1	696.96	**<0.001**	1	241.29	**<0.001**	1	212.80	**<0.001**
Salinity (S)	–	–	–	3	108.36	**<0.001**	3	71.02	**<0.001**	3	29.85	**<0.001**	3	212.47	**<0.001**	3	234.43	**<0.001**
Seed morph (SM)	1	23.73	**<0.001**	1	8.87	**0.003**	1	0.00	0.949	1	1.14	0.287	1	0.08	0.782	1	0.28	0.598
P × S	–	–	–	3	54.25	**<0.001**	3	14.21	**<0.001**	3	5.57	**0.001**	3	19.33	**<0.001**	3	27.11	**<0.001**
P × SM	1	0.15	0.697	1	0.14	0.710	1	0.43	0.512	1	0.35	0.553	1	6.74	**0.010**	1	14.75	**<0.001**
S × SM	–	–	–	3	0.65	0.586	3	0.24	0.869	3	0.46	0.709	3	3.55	**0.016**	3	6.16	**0.001**
P × S × SM	–	–	–	3	2.63	0.052	3	2.79	**0.043**	3	1.74	0.162	3	2.30	0.080	3	3.06	**0.030**
Error	36			144			144			144			144			144		

		**Total mass**			**Seed ratio**			**Seed size**						**Germination**				
								**Brown seed**			**Black seed**			**Brown seed**			**Black seed**	
**Source**	**df**	***F*-value**	***P*-value**	**df**	***F*-value**	***P*-value**	**df**	***F*-value**	***P*-value**	**df**	***F*-value**	***P*-value**	**df**	***F*-value**	***P*-value**	**df**	***F*-value**	***P*-value**

Population (P)	1	88.08	**<0.001**	1	194.88	**<0.001**	1	921.71	**<0.001**	1	1891.30	**<0.001**	1	0.01	0.907	1	92.31	**<0.001**
Salinity (S)	3	288.21	**<0.001**	3	24.97	**<0.001**	3	169.07	**<0.001**	3	132.89	**<0.001**	3	0.27	0.845	3	37.02	**<0.001**
Seed morph (SM)	1	0.01	0.930	1	4.39	**0.038**	1	10.85	**0.001**	1	3.97	**0.048**	1	1.67	0.202	1	0.36	0.551
P × S	3	20.09	**<0.001**	3	16.76	**<0.001**	3	4.13	**0.008**	3	8.90	**<0.001**	3	3.04	**0.038**	3	7.12	**<0.001**
P × SM	1	11.78	**0.001**	1	10.73	**0.001**	1	0.03	0.854	1	0.04	0.844	1	0.12	0.726	1	9.01	**0.004**
S × SM	3	5.16	**0.002**	3	0.35	0.790	3	0.93	0.430	3	5.17	**0.002**	3	4.92	**0.005**	3	0.82	0.488
P × S × SM	3	2.93	**0.036**	3	0.65	0.583	3	2.13	0.099	3	13.72	**<0.001**	3	5.51	**0.002**	3	4.98	**0.004**
Error	144			144			144			144			48			48		

*–Addition of salt was not begun during seedling stage. Significant values are highlighted in bold.*

### Vegetative and Reproductive Traits of Plants (F_1_) Grown in Common Environment Experiment

**FIGURE 4:**
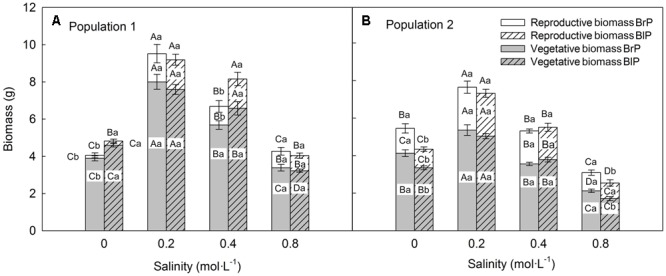
Effects of soil salinity and seed morph on biomass allocation (mean ± 1 SE) of F_1_ (filial generation 1) plants of the two populations of *Suaeda corniculata*. Different letters above bars indicate significant differences (*P* < 0.05) in total biomass and different letters in bars significant differences (*P* < 0.05) in vegetative or reproductive biomass. Uppercase letters indicate differences across all salinity levels in the same seed morph and lowercase letters differences between seed morphs for the same salinity level. BrP, plants from brown seeds; BlP, plants from black seeds.

Biomass was significantly affected by population and salinity but not by seed morph (**Table 2**). Plants produced the most biomass under 0.2 mol⋅L^-1^ salinity, and the total, vegetative and reproductive mass decreased with a further increase in salinity (**Figures 4A,B**). Reproductive allocation (reproductive mass/total biomass) increased with level of salinity. From 0 to 0.8 mol⋅L^-1^, reproductive allocation increased 16% for P1 and 12% for P2. In addition, plants had the lowest reproductive allocation under 0 mol⋅L^-1^ (4% for P1 vs. 24% for P2).

Total plant biomass of P1 was larger than that of P2, which mostly was due to P1 having a larger vegetative mass than P2. However, P1 produced fewer seeds (less reproductive mass) than P2 (**Figures 4A,B**), which resulted in lower reproductive allocation in P1 than in P2.

### Traits of Seeds Produced in Common Environment Experiment

**FIGURE 5:**
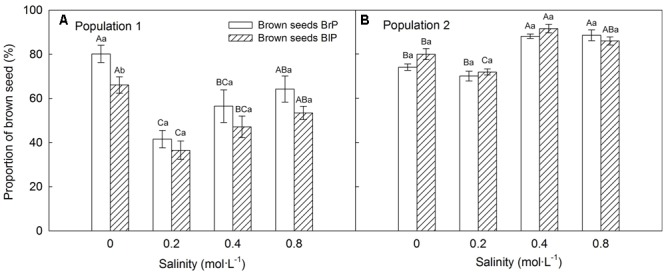
Effects of soil salinity and F_1_ (filial generation 1) seed morph on brown seed proportion (mean ± 1 SE) produced by F_1_ experimental plants of *Suaeda corniculata.* Different uppercase letters indicate differences (*P* < 0.05) in brown seed proportion across all salinity levels in the same seed morph and lowercase letters differences (*P* < 0.05) in brown seed proportion between seed morphs for the same salinity level. BrP, plants from brown seeds; BlP, plants from black seeds.

Plants grown under 0.2 mol⋅L^-1^ salinity produced the greatest number of seeds, and seed number decreased with an increase in salinity (Supplementary Figure S1). The proportion of seed morphs produced by the plants was significantly influenced by population, salinity and morph of the maternal seeds from which plants were grown (**Table 2**). The brown seed proportion produced by P1 plants was lower than that produced by P2 plants (**Figures 5A,B**). The proportion of brown seeds was lowest under 0.2 mol⋅L^-1^ salinity and increased with an increase in salinity (**Figures 5A,B**). For plants grown under 0.2 to 0.8 mol⋅L^-1^, the proportion of brown seeds increased 19.8% for P1 and 16.2% for P2 (**Figures 5A,B**).

Diameter of the two seed morphs produced by the experimental plants was significantly affected by population, salinity and maternal seed morph (**Table 2**). For both seed morphs, seeds of P1 were smaller than those of P2 (Supplementary Figure S2). Both brown and black seeds were larger when their maternal plants were grown in 0.2 mol⋅L^-1^ or 0.4 mol⋅L^-1^, whereas plants grown in 0 mol⋅L^-1^ produced the smallest seeds (Supplementary Figure S2).

**FIGURE 6:**
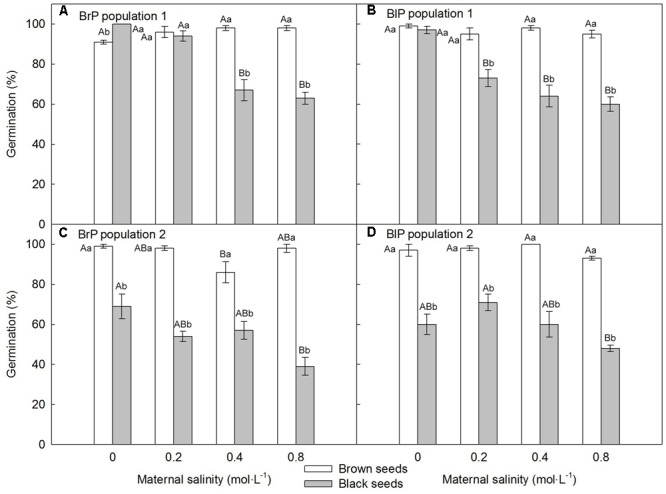
Germination percentage (mean ± 1 SE) of fresh F_2_ (filial generation 2, see **Figure 2**) dimorphic seeds produced by F_1_ (filial generation 1) experimental plants of *Suaeda corniculata.* Different uppercase letters indicate differences (*P* < 0.05) across all salinity levels in the same seed morph and different lowercase letters differences (*P* < 0.05) between seed morphs for the same maternal salinity level. BrP, plants from brown seeds; BlP, plants from black seeds.

For both populations, brown seeds germinated to a high percentage (>85%) in all maternal salinity concentrations (**Figures 6A,C**), whereas germination of black seeds differed between populations and maternal salinities (**Table 2**). Germination percentage of black seeds was higher for P1 than for P2. Black seeds of P1 germinated to 100% only in 0 mol⋅L^-1^ maternal salinity, whereas germination of black seeds of P2 reached only about 70% (**Figures 6B,D**). Maternal salt stress reduced germination of black seeds (**Figures 6A–D**).

## Discussion

The present study asked if there are phenotypic differences in life history traits between two populations of *Suaeda corniculata*. To minimize carryover effects from field environments, plants from the two seed morphs derived from field populations were grown in a common environment. Distinct phenotypic differences between populations in the field were retained in the common environment. Indeed, seed morph ratio and germination percentage of dimorphic seeds differed greatly between magnitudes of soil salinity. Although many studies have tested the effects of soil salinity on a limited number of plant life history traits (Yao et al., 2010; Wang et al., 2012, 2015), our study appears to be the first to clearly detail the phenotypically plastic response of heteromorphic seeds to different levels of salinity by cultivating plants from different geographic populations in a common environment.

Soil salinity in arid and semi-arid zones has been considered both an extreme environmental stress and a source of disturbance to plants (Ungar, 1978; Khan and Ungar, 2001; Imbert, 2002), and halophytes have morphological and physiological adaptations that facilitate their survival and growth under such harsh conditions (Debez et al., 2004; Yang et al., 2010). Plants of the seed-dimorphic species *Suaeda splendens* grew better at 0.2 mol⋅L^-1^ NaCl than in pure water (Redondo-Gómez et al., 2008), and the optimum growth of *Salicornia dolichostachya* was at 0.3 mol⋅L^-1^ NaCl (Katschnig et al., 2013). Likewise, in the present study F_1_ plants of *S. corniculata* derived from both seed morphs performed better at low salinity than in salt-free tap water. And although a high level of soil salinity had adverse effects on plant growth and seed production, the plants allocated a higher proportion of biomass to seeds under salinity stress. Stress can cause a shift in the proportion of biomass allocated to vegetative and reproductive components (Lu et al., 2012). These plastic responses in life history traits to stress can be interpreted as to be adaptive because they increase the seed production component of fitness (Primack and Kang, 2005; Lu et al., 2016).

Studies have shown that growth conditions of the mother plant can alter the proportion of different seed morphs produced by an individual plant (reviewed in Baskin and Baskin, 2014), but the results are inconsistent. For example, seed morph ratio was not changed by growth of maternal plants of *Suaeda aralocaspica* (Amaranthaceae) in different salinities (Wang et al., 2012), whereas high salt stress increased the proportion of (non-dormant) brown seeds of *Chenopodium album* (Amaranthaceae) (Yao et al., 2010) and of *Suaeda salsa* (Amaranthaceae) (Wang et al., 2015). In our study, the proportion of brown seeds increased with increased salinity levels in the two populations of *S. corniculata*, thus agreeing with the results of Yao et al. (2010) and Wang et al. (2015). Because they germinate to high percentages in a wide range of salinities, brown seeds of Amaranthaceae species have been considered to be more fit than black seeds in harsh saline habitats (Khan et al., 2001; Song et al., 2008; Yao et al., 2010; Wang et al., 2015). However, Cao et al. (2012) showed that brown seeds of *S. corniculata* lost viability easily after exposure to salt stress, whereas black seeds did not. Thus, the ecological significance of brown versus black seeds is highly complex and should not be determined only by germination percentage. Since brown seeds are larger and growth of seedlings derived from them is faster than for black seeds, seedlings may benefit from more resources stored in large brown seeds (Grieve and Francois, 1992; Jakobsson and Eriksson, 2000; Easton and Kleindorfer, 2009), which would then lead to faster establishment. Thus, brown seed cohorts may benefit from exploiting temporarily favorable conditions, for example high soil moisture (and thus lower salinity) after rainfall. In which case, they may establish populations rapidly in saline habitats. On the other hand, black seeds of *S. corniculata* cannot germinate at high salinity, but they can form a persistent seed bank, which enables them to persist in the habitat should the entire population die before reproducing in a particular year. Black seed cohorts mainly germinate in the summer rainy season (Cao et al., 2012), and consequently they may be exposed to less harsh conditions for establishment than brown seed cohorts.

Variation in morph ratio and seed size has been found among populations of diaspore-heteromorphic species. For the annual halophyte, *Suaeda salsa* (Amaranthaceae), brown/black seed ratio and 100-seed mass were significantly higher in the intertidal zone (4.5 and 3.3 g⋅kg^-1^, Na^+^ and Cl^-^, respectively) than in an inland saline habitat (2.4 and 2.0 g⋅kg^-1^, Na^+^ and Cl^-^, respectively) (Song et al., 2008). The proportion of brown seeds and reserve mass (embryo plus perisperm) of black seeds of *Chenopodium album* were higher in populations on flat ground than that of a population growing on a slope (Yao et al., 2010). However, whether these differences between populations were due to genetics (G), environment (E) or G x E interactions was not determined. We conducted a common environment experiment in which F_1_ plants were grown from F_1_ seeds under different salinity levels to distinguish possible genetic differentiation between populations and to demonstrate phenotypic plastic response to different salt concentrations. For *S. corniculata*, the field investigation and common environment experiments revealed that the differences between populations were significant in both morph ratio and seed size, as well as high salinity increased brown seed proportion but had limited impact on seed size. Quinn and Colosi (1977) suggested that one generation of seed production under the same conditions is required to demonstrate that differences in germination are genetically based. However, we used F_1_ plants for testing post-germination life history traits, and thus the possibility exists that differences among the traits could have been due to some carryover effects on plants grown from F_1_ seeds (Whittle et al., 2009; Herman et al., 2012; Yang et al., 2015b).

Seed dimorphism in *S. corniculata* may be a bet-hedging strategy since the offspring have two distinct life histories to cope with a variable environment (Venable, 1985a; Simons, 2011). As such, then, it seems that population persistence is more likely for plants producing both brown and black seeds than it would be for them producing either one or the other seed morph. However, to document bet-hedging and thus to understand the ecological consequences of production of dimorphic seeds, a comparative life history/demographic analysis of each of the two morphs is required (Venable, 1985a,b; Venable and Levin, 1985). It must be shown that the two morphs maximize the geometric mean of the number of offspring (Ro, a measure of fitness) across generations (Simons, 2011) in order to prove that the seed heteromorphism in *S. corniculata* is a bet-hedging strategy.

## Conclusion

According to the field investigation and common environment experiments, phenotypic differences between *S. corniculata* populations are genetically based. Adverse effects of salinity stress are alleviated by the ability of plants to allocate a high proportion of resources to reproduction. Morph production and germination behavior of dimorphic seeds are influenced by the level of soil salinity, suggesting that soil salinity plays an ecologically important role in population regeneration of *S. corniculata* in the natural habitat.

## Author Contributions

ZH, FY, CB, JB, and XY conceived and designed the experiments. FY, DC, and XY performed the experiments and analyzed the data. FY, ZH, CB, and JB wrote the manuscript.

## Conflict of Interest Statement

The authors declare that the research was conducted in the absence of any commercial or financial relationships that could be construed as a potential conflict of interest.
