# Supplementary material for: Divergence in Life History Traits between Two Populations of a Seed-Dimorphic Halophyte in Response to Soil Salinity
Source: Front Plant Sci. 2017 Jun 16;8:1028. doi: 10.3389/fpls.2017.01028 (PMC5472680; doi:10.3389/fpls.2017.01028)

**Figure S1**. Effects of soil salinity and F_0_ seed morph on number of seeds (mean± 1 S.E.) of the two seed morphs produced by F_1_ experimental plants of *Suaeda corniculata.* Different uppercase letters indicate differences (*P* < 0.05) in total number of seeds across all salinity levels in the same seed morph and lowercase letters differences (*P* < 0.05) in number of seeds between seed morphs for the same salinity level. BrP, plants from brown seeds; BlP, plants from black seeds.


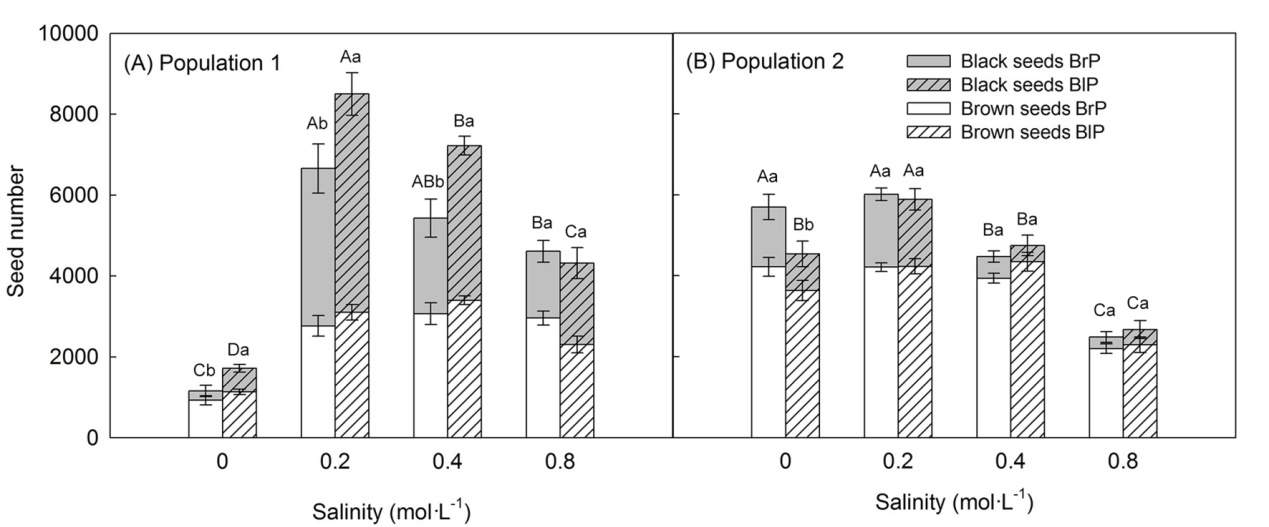


**Figure S2**. Seed diameter (mean ± 1 S.E.) of F_2_ offspring produced by F_1_ experimental plants of *Suaeda corniculata.* Different uppercase letters indicate differences (*P* < 0.05) across all salinity levels in the same seed morph and lowercase letters differences (*P* < 0.05) between seed morphs for the same salinity level. BrP, plants from brown seeds; BlP, plants from black seeds.


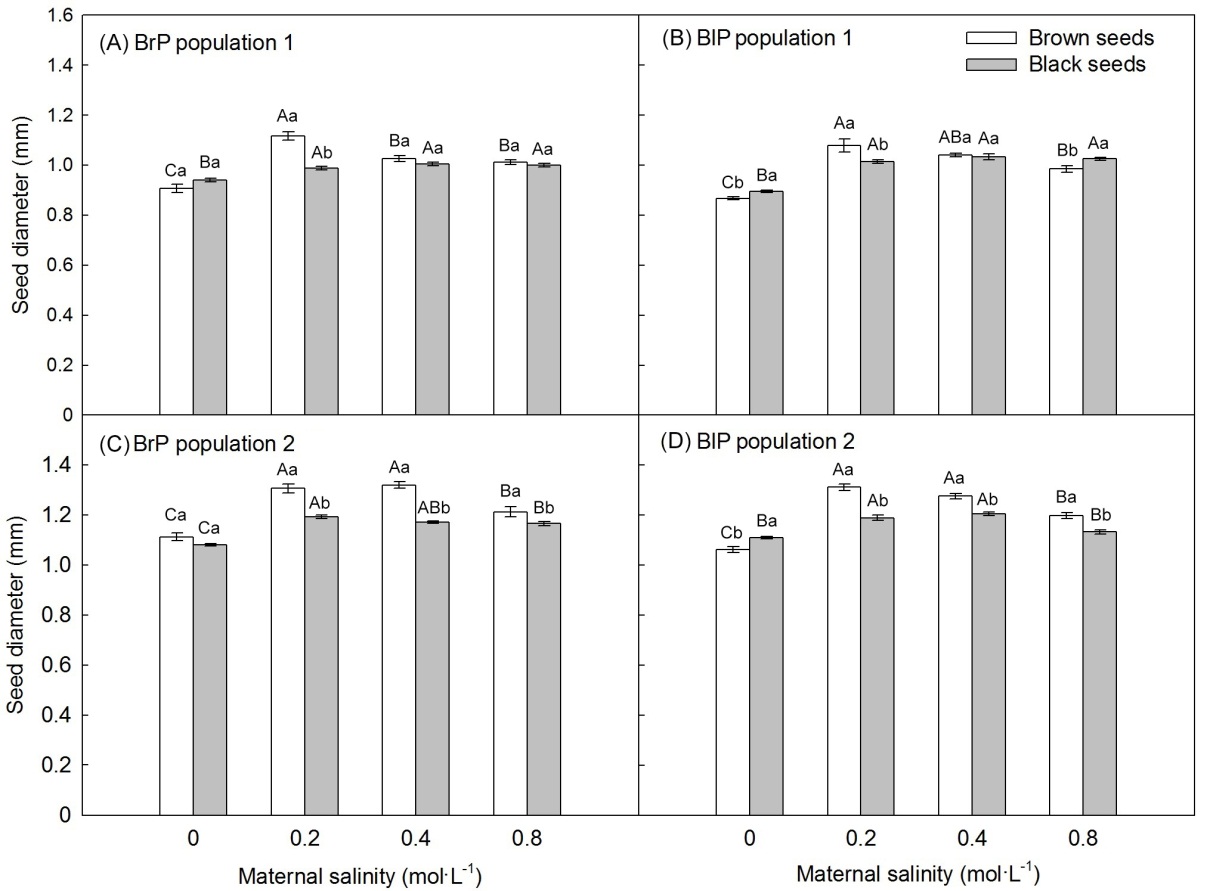

Supplement: Supplementary file 1 [file Data_Sheet_1.DOCX]
